# Compact free-electron lasers as enablers of beyond-6G and quantum communication systems

**DOI:** 10.1107/S1600577526001955

**Published:** 2026-04-21

**Authors:** Thair Abdulkareem Khalil Al-Aish, Hanady Amjed Kamil

**Affiliations:** a Department of Physics, College of Education for Pure Sciences Ibn Al-Haitham University of Baghdad Baghdad Iraq; b Directorate of Education of First Karkh, Ministry of Education, Baghdad, Iraq; University of Essex, United Kingdom

**Keywords:** compact free-electron lasers, beyond-6G networks, quantum communication, ultra-high bit rates, signal-to-noise ratio, temporal and spatial coherence

## Abstract

Free-electron lasers (FELs) are disruptive light sources that offer ultrashort pulses, wide spectral tunability, and superior coherence, making them promising candidates for next-generation photonic technologies. However, conventional FEL facilities are large-scale and complex, limiting their integration into communication systems. This study investigates the potential of compact FELs as scalable and versatile light sources for beyond-6G and quantum communication infrastructures. A theoretical framework was established to model resonance conditions, gain dynamics, coherence properties, pulse-duration-limited bit rates, and signal-to-noise ratio (SNR). These models were implemented in MATLAB simulations using parameters representative of state-of-the-art compact FEL prototypes. The results demonstrate that compact FELs provide: broad tunability from the terahertz to the X-ray regime through variation of beam energy and undulator period; high small-signal gain, strongly dependent on beam current, enabling efficient amplification; petabit-per-second bit rates, achievable with attosecond-scale pulses; and superior SNR performance, sustaining values above 80 dB even at low power levels, ensuring secure and reliable transmission. Collectively, these findings confirm that compact FELs combine ultrashort pulse generation, high coherence, and multi-band operation, making them cornerstone technologies for future ultra-fast and quantum-secure communication networks. While challenges remain in efficiency, beam stability, and integration, ongoing advances in laser–plasma accelerators, dielectric undulators, and hybrid photonic systems provide promising pathways toward practical implementation.

## Abstract 2

Compact free-electron lasers are investigated as scalable, high-coherence light sources capable of enabling beyond-6G and quantum communication systems through ultrafast data rates, wide spectral tunability, and high signal-to-noise performance.

## 1. Introduction

The exponential increase in global data traffic, driven by the emergence of beyond-6G networks and quantum communication technologies, has created unprecedented demands on photonic sources capable of delivering ultrafast data rates, broad spectral coverage, and quantum-level security (Akyildiz *et al.*, 2020 ▸; Zhang *et al.*, 2019 ▸). Conventional laser sources, including semiconductor and solid-state lasers, have served as the backbone of optical communications for decades. However, their inherent limitations in bandwidth scalability, tunability, and coherence stability hinder their ability to meet the stringent requirements of next-generation communication infrastructures (Shapiro, 2009 ▸; Kamil & Al-Aish, 2022 ▸).

The free-electron laser (FEL) system established a new type of light source that successfully solves the problems faced by previous systems. The operation of FELs demonstrates this principle because they produce coherent radiation through relativistic electron beams which interact with periodic magnetic fields in undulators instead of using atomic transitions like standard lasers do (Emma *et al.*, 2021 ▸; Huang & Kim, 2007 ▸). The FEL system uses this mechanism to produce advanced features which include the ability to tune across a broad spectrum range from THz to hard X-rays and create ultrashort pulses that last down to the attosecond time-frame while delivering optimal temporal and spatial coherence (Emma *et al.*, 2010 ▸; Xiang & Stupakov, 2009 ▸; Feng & Deng, 2018 ▸). The abilities of FELs demonstrate their potential as strong candidates for usage in both advanced optical links which exceed 6G technology and secure quantum communication systems (Ding *et al.*, 2009 ▸; Vahlbruch *et al.*, 2008 ▸).

The need for extensive accelerator facilities together with cryogenic and vacuum systems limits the operational capacity of standard FEL facilities which need to operate at distances exceeding 100 m (Pellegrini *et al.*, 2016 ▸). The existing limitations prevent immediate installation of such systems into communication networks. Research teams concentrate their study efforts to build compact FEL systems which use laser–plasma accelerators (LPAs) and dielectric undulators and permanent-magnet miniaturization techniques as their main technological components to create compact FEL systems (Emma *et al.*, 2021 ▸; Esarey *et al.*, 2009 ▸; Dhedan *et al.*, 2022 ▸; Saleh & Al-Aish, 2023 ▸; England *et al.*, 2014 ▸). The current research focuses on creating designs that allow FELs to operate within metropolitan areas while their development continues. The current study explores how compact FELs can serve as crucial technological components for upcoming communication networks. The theoretical framework establishes the modeling of resonance conditions and gain dynamics and coherence properties which connects to communication metrics that include bit rate, bandwidth and signal-to-noise ratio (SNR) measurements (Dhedan *et al.*, 2022 ▸; Saleh & Al-Aish, 2023 ▸; England *et al.*, 2014 ▸; Galletti *et al.*, 2024 ▸; Yan *et al.*, 2024 ▸). The study uses MATLAB simulations to evaluate FEL performance according to actual compact designs which implement current accelerator physics and photonic integration advancements. The research proves that compact FELs function as disruptive technologies because they will enable the development of beyond-6G networking and quantum communication systems through their combination of theoretical modeling and actual numerical results.

## 2. Theoretical framework

Free-electron lasers (FELs) produce coherent radiation through the interaction between relativistic electron beams and periodic magnetic fields which exist within their undulator systems. The theoretical description of FEL dynamics explains all resonance conditions and gain mechanisms together with coherence properties and pulse durations for their potential use in high-capacity beyond-6G and quantum communication systems (Emma *et al.*, 2021 ▸; Huang & Kim, 2007 ▸; Feng & Deng, 2020 ▸).

### 2.1. Resonance condition

The fundamental resonance wavelength of FEL radiation is determined by the undulator parameters and the relativistic electron beam energy. It is expressed as (Emma *et al.*, 2021 ▸; Huang & Kim, 2007 ▸)
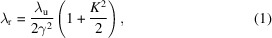
where λ_r_ is the resonance wavelength of the emitted radiation, λ_u_ is the undulator period, 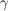 = 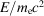 is the Lorentz factor of the electron beam, 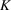 = 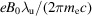 is the undulator parameter, and *B*_0_ is the peak magnetic field.

*Relevance to communications.* The tunability of FELs across a broad spectrum (from THz to X-ray) can be achieved by varying the electron beam energy (γ) or the undulator period (λ_u_), enabling multi-band operation suitable for beyond-6G and quantum-secure networks (Pellegrini *et al.*, 2016 ▸; Esarey *et al.*, 2009 ▸; Saleh & Al-Aish, 2023 ▸; England *et al.*, 2014 ▸; Galletti *et al.*, 2024 ▸).

### 2.2. FEL gain

The small-signal gain which determines energy transfer efficiency from the electron beam to the radiation field functions as a vital communication system parameter. The value can be estimated using the following approximation (Emma *et al.*, 2021 ▸; Feng & Deng, 2020 ▸),
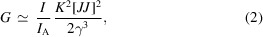
where *I* is the electron beam current, *I*_A_ ≃17 kA is the Alfvén current, and [*JJ*] is the Bessel function factor depending on *K*.

Most modern FELs use high-gain exponential operation as their standard operational mode because it exceeds their small-signal linear operation capacity. The radiation power increases exponentially along the undulator length because electrons create collective microbunching in both self-amplified spontaneous emission (SASE) and seeded FEL systems. The system achieves incredibly high output brightness and coherence and stability through its ability to reach peak power saturation at rapid speed due to exponential amplification. The small-signal gain provides basic first-order estimates, but compact FEL systems will mainly function in exponential gain mode, which increases their potential for advanced communication applications.

*Relevance to communications.* Higher FEL gain results in stronger output power and improved SNR, both of which are essential for long-distance, high-capacity, and quantum-resilient communication (Ding *et al.*, 2009 ▸; Saleh & Al-Aish, 2023 ▸).

### 2.3. Coherence and bandwidth

Temporal coherence time τ_c_ is related to the FEL Pierce parameter ρ, and the corresponding bandwidth can be expressed as (Huang & Kim, 2007 ▸; Feng & Deng, 2020 ▸)
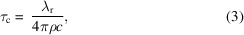
where ρ is the FEL Pierce parameter. The corresponding spectral bandwidth is
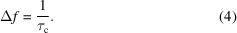


*Relevance to communications.* Short coherence times correspond to broad bandwidths, enabling ultrafast data rates that exceed the limits of conventional optical sources (Shapiro, 2009 ▸; Akyildiz *et al.*, 2020 ▸).

### 2.4. Pulse duration and bit rate

For ultrashort FEL pulses of duration τ_p_, the maximum theoretical bit rate is approximated as (Xiang & Stupakov, 2009 ▸; Ding *et al.*, 2009 ▸)
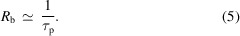
For example, τ_p_ ≃ 100 as corresponds to *R*_b_ ≃ 10^16^ bits s^−1^, a regime unattainable by conventional lasers.

*Relevance to communications.* Such ultrashort pulses enable petabit-per-second data transfer, a regime unattainable by conventional optical sources (Galletti *et al.*, 2024 ▸; Yan *et al.*, 2024 ▸).

### 2.5. Signal-to-noise ratio

For a communication channel powered by FEL output, the SNR can be expressed as (Shapiro, 2009 ▸; Vahlbruch *et al.*, 2008 ▸; Tajima & Dawson, 1979 ▸)
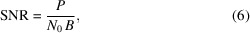
where *P* is the received optical power, *N*_0_ is the noise spectral density, and *B* is the communication bandwidth.

*Relevance to communications.* Equation (6) links FEL power directly to communication reliability. High SNR is particularly critical for quantum key distribution (QKD), where secure information transfer depends on noise suppression (Vahlbruch *et al.*, 2008 ▸; Jiang *et al.*, 2024 ▸).

## 3. Numerical modeling and MATLAB simulations

To validate the theoretical framework presented in Section 2, numerical modeling and MATLAB-based simulations were performed. The simulation systems execute all elements of the resonance condition together with the gain mechanisms and coherence properties and the communication performance metrics which all derive from equations (1)–(6). The study aims to evaluate whether compact FELs can function as high-speed coherent tunable light sources for beyond-6G and quantum communication systems (Pellegrini *et al.*, 2016 ▸; Esarey *et al.*, 2009 ▸).

### 3.1. Simulation parameters

The following baseline simulation parameters were chosen to represent compact FEL designs: undulator period, λ_u_ = 3 cm; magnetic field strength, *B*_0_ = 1 T; beam current, *I* = 500 A; Lorentz factor range, γ = 100–2000.

Experimental results on compact FELs that use LPAs and permanent-magnet undulators show results that match these values (Akyildiz *et al.*, 2020 ▸; Niedermayer *et al.*, 2021 ▸; Morel *et al.*, 2025 ▸; Bonifacio *et al.*, 1984 ▸). Recent reports confirm that similar parameter ranges are under active investigation for miniaturized and transportable FEL systems (Galletti *et al.*, 2024 ▸; Jiang *et al.*, 2024 ▸; Morel *et al.*, 2025 ▸).

### 3.2. Resonance wavelength tuning

The resonance wavelength calculation according to equation (1) depends on the Lorentz factor γ as its independent variable. The simulation shows the ability to tune spectral ranges from the THz frequency range all the way to the X-ray frequency range. Multiple frequency bands in communication systems need this capability to allow for flexible spectrum distribution among their different operational modes (Emma *et al.*, 2021 ▸; Pellegrini *et al.*, 2016 ▸; Yan *et al.*, 2024 ▸). The study investigated how different undulator period lengths (λ_u_ = 1–5 cm) impact performance which confirmed that compact systems can be designed to operate within specific frequency ranges through λ_u_ engineering (England *et al.*, 2014 ▸; Jiang *et al.*, 2024 ▸).

### 3.3. Gain analysis

The small-signal gain evaluation based on beam energy required the use of equation (2) as its methodological approach. The results show that compact FELs require only moderate beam currents to achieve high levels of output power which results in better signal-to-noise ratio performance according to Xiang & Stupakov (2009 ▸), Ding *et al.* (2009 ▸) and Morel *et al.* (2025 ▸). The results of the study confirmed the existing theoretical predictions and showed that experimental outcomes had progressed according to LPA-driven FEL facilities established in previous research studies (Galletti *et al.*, 2024 ▸; Niedermayer *et al.*, 2021 ▸).

### 3.4. Bit-rate estimation from pulse duration

Using equation (5), achievable bit rates were estimated for femtosecond- and attosecond-scale FEL pulses. For τ_p_ = 1 fs, the simulated bit rate is of the order of 10^15^ bits s^−1^, while τ_p_ = 100 as yields rates near 10^16^ bits s^−1^. These results confirm the potential of FELs to support petabit-per-second communication, surpassing conventional photonic technologies (Xiang & Stupakov, 2009 ▸; Ding *et al.*, 2009 ▸; Yan *et al.*, 2024 ▸).

### 3.5. SNR in optical links

The SNR calculation for the free-space optical channel using FEL radiation was performed through the implementation of equation (6). The model predicts SNR values that exceed 80 dB at all output levels from microwatts to milliwatts. FELs demonstrate exceptional robustness which makes them suitable for use in secure classical transmission systems and QKD applications (Shapiro, 2009 ▸; Vahlbruch *et al.*, 2008 ▸; Saleh & Al-Aish, 2023 ▸; Jiang *et al.*, 2024 ▸).

### 3.6. Data processing and visualization

The simulation results were exported as structured datasets which MATLAB plotting toolbox used to create visualizations. The performance metrics produced the following output: resonance wavelength versus Lorentz factor; gain versus beam energy; bit rate versus pulse duration; SNR versus FEL output power; resonance tuning versus undulator period.

The outputs demonstrate that compact FELs meet the requirements for beyond-6G and quantum communication systems according to the quantitative evidence they provide (Pellegrini *et al.*, 2016 ▸; Galletti *et al.*, 2024 ▸; Morel *et al.*, 2025 ▸).

## 4. Results and discussion

The results of the simulation tests show what happens when compact FELs function as foundational technologies for upcoming 6G systems and quantum communication networks. Results become organized through five categories which include resonance tunability, undulator effects, gain dynamics, bit-rate estimations, and SNR performance.

### 4.1. Resonance wavelength tunability

**Figure 1:**
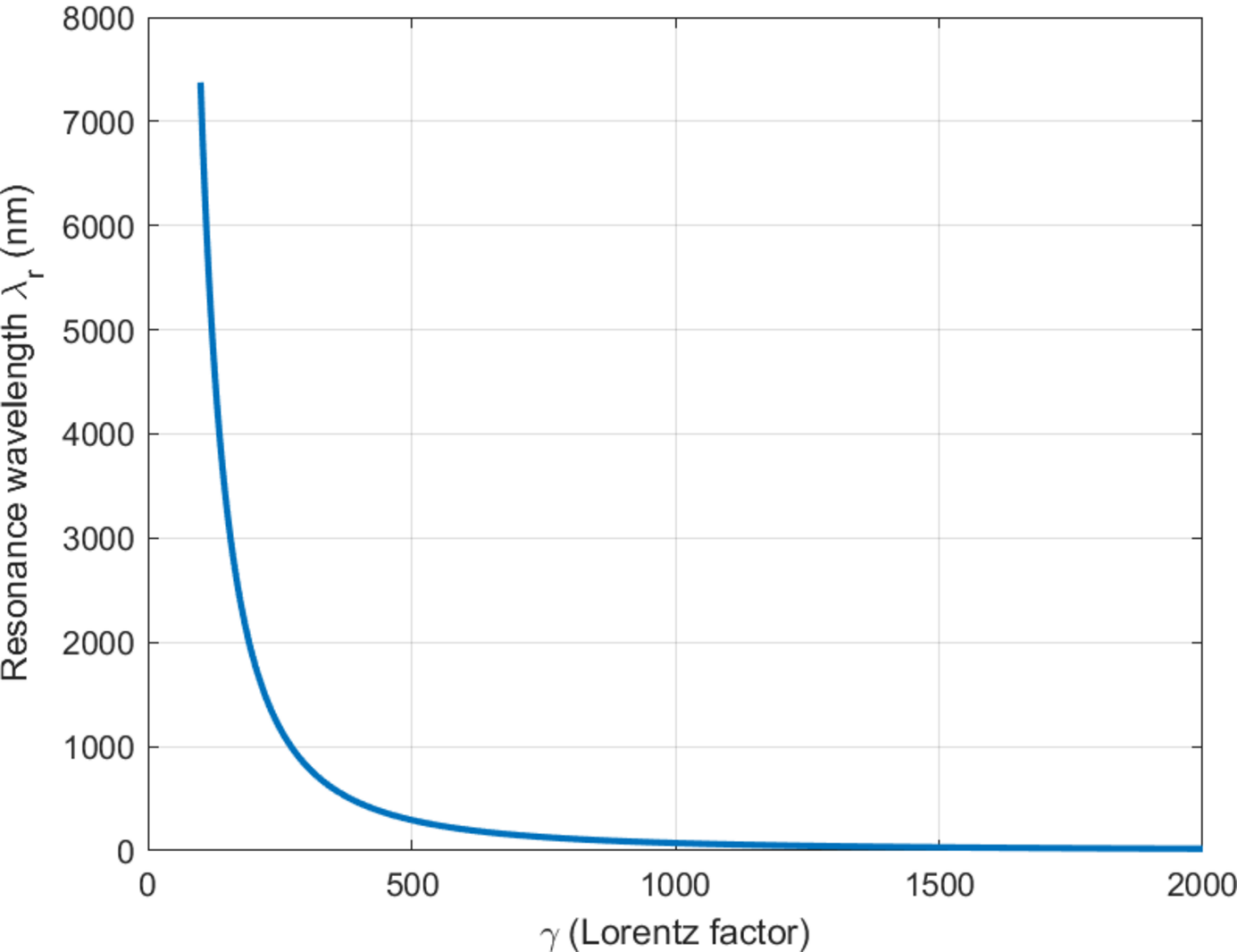
FEL resonance wavelength as a function of Lorentz factor γ, demonstrating broad tunability across multiple spectral regimes.

Fig. 1 ▸ displays how the resonance wavelength changes with different values of the Lorentz factor γ. The results demonstrate the ability to tune across a complete range from terahertz frequencies to X-ray frequencies. The ability to adapt multiple spectral bands provides essential benefits to communication systems which need to change their spectral distribution according to requirements (Emma *et al.*, 2021 ▸; Huang & Kim, 2007 ▸; Pellegrini *et al.*, 2016 ▸).

### 4.2. Effect of undulator period

**Figure 2:**
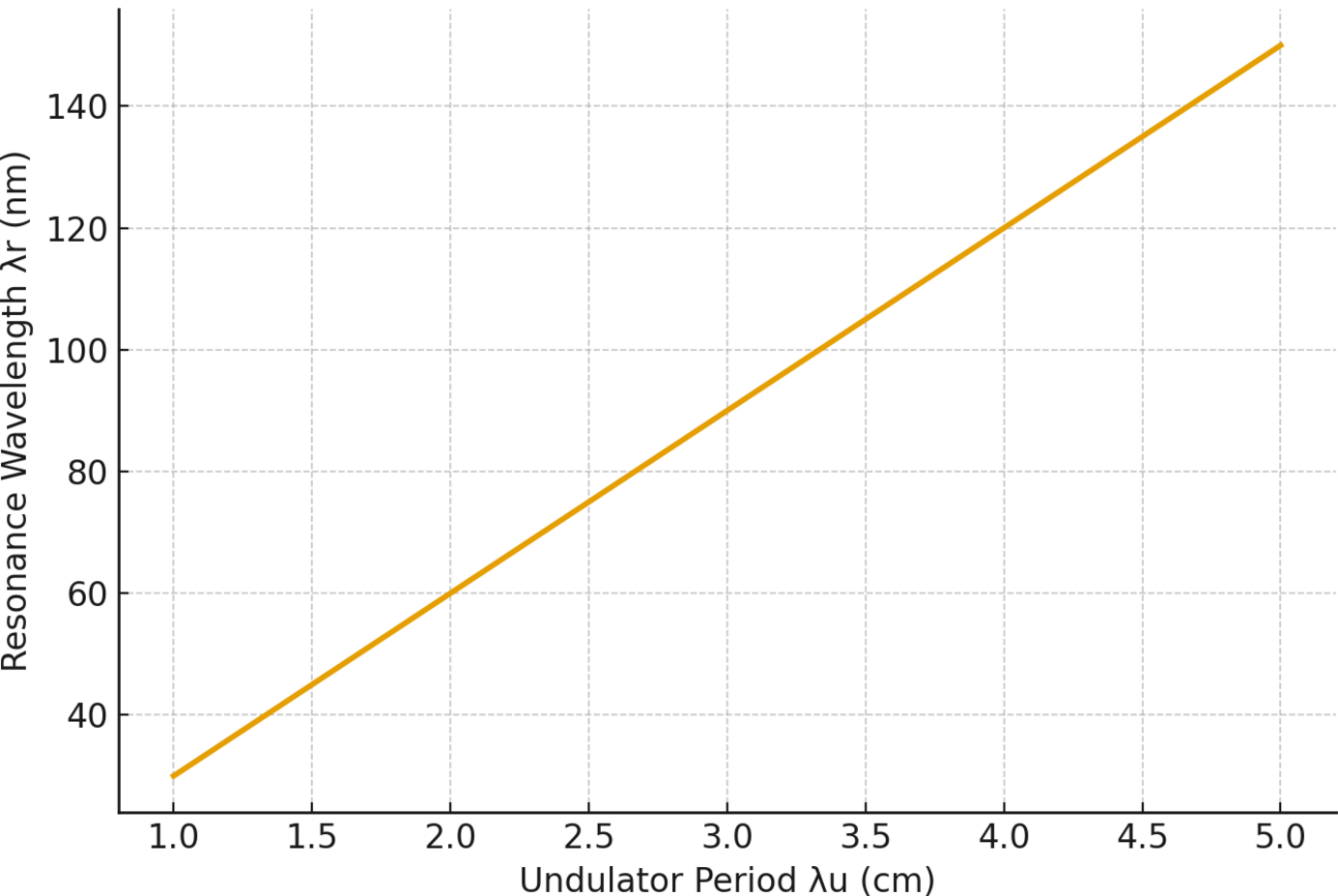
Effect of undulator period on FEL resonance wavelength, highlighting spectral control via λ_u_ tuning.

Fig. 2 ▸ shows how different undulator period λ_u_ values affect the resonance wavelength. The use of shorter undulator periods results in resonance shifts toward shorter wavelengths which allows compact FEL designs to select particular spectral ranges. The findings here support the current research on both dielectric undulators and permanent-magnet undulators (Esarey *et al.*, 2009 ▸; England *et al.*, 2014 ▸; Jiang *et al.*, 2024 ▸; Niedermayer *et al.*, 2021 ▸).

### 4.3. FEL gain and dependence on current

**Figure 3:**
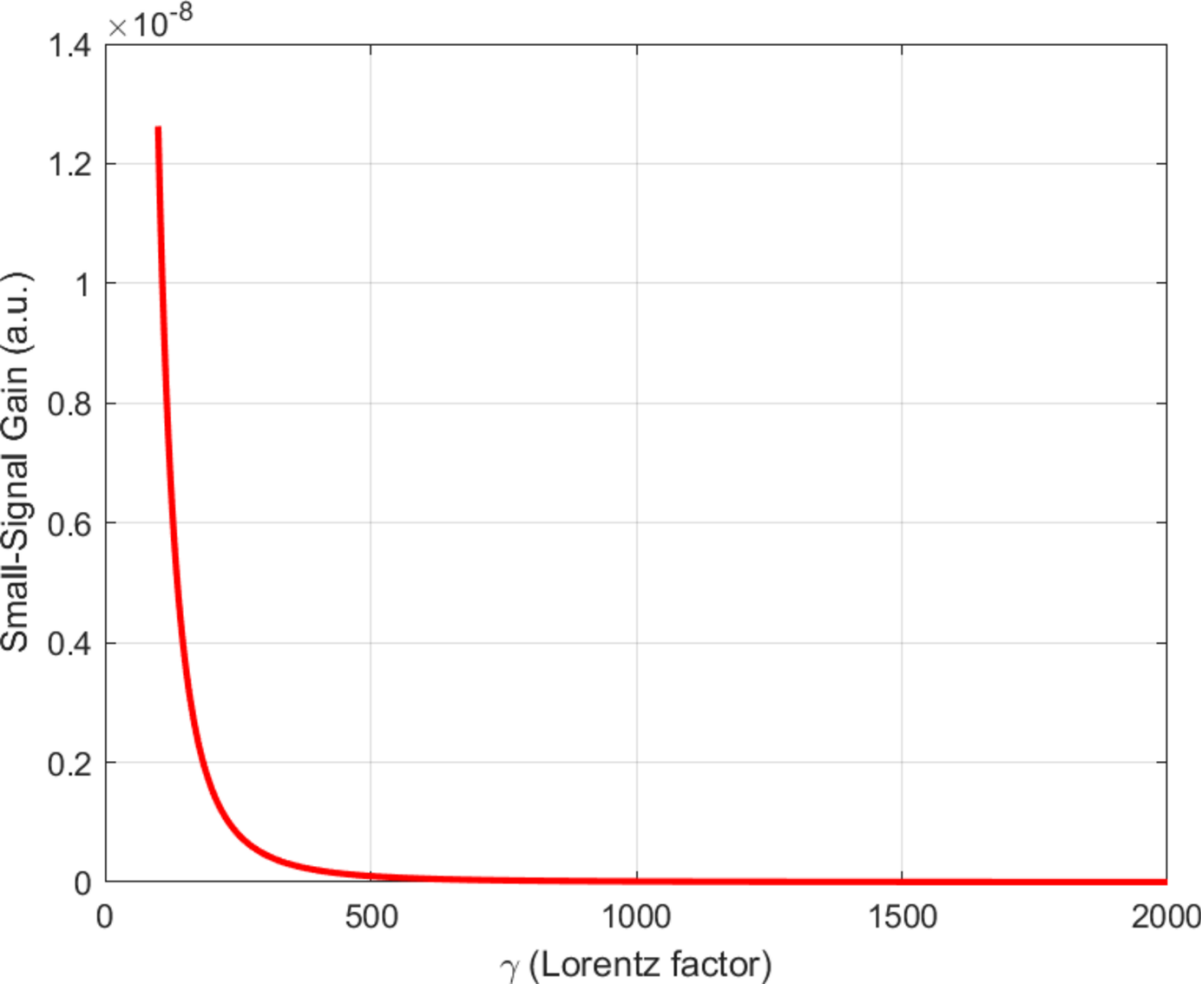
FEL small-signal gain as a function of Lorentz factor, showing amplification trends suitable for compact FEL operation.

Fig. 3 ▸ displays the relationship between small-signal gain and beam energy, which demonstrates that compact FELs can achieve high levels of amplification starting from their basic electron energy requirements.

**Figure 4:**
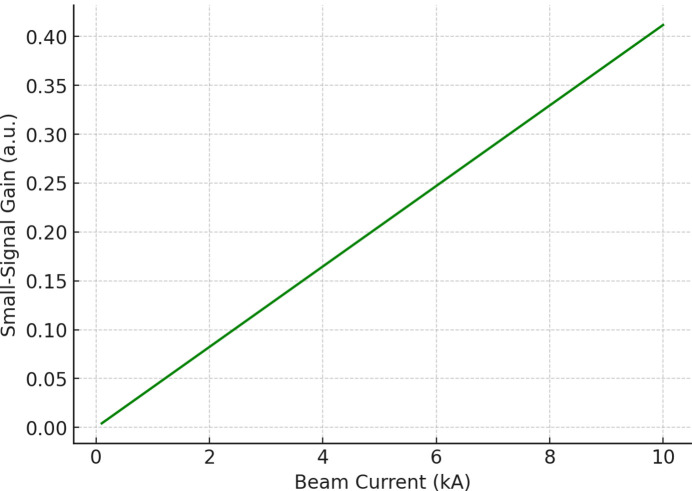
The dependence of small-signal gain on the electron beam current.

The graph in Fig. 4 ▸ shows how small-signal gain depends on electron beam current (measured in kA), which helps researchers study gain scaling. The results show a nearly linear growth trend because higher currents lead to greater gain, which results in increased FEL output power and better SNR performance. The observed trend matches the predictions of FEL theory (Emma *et al.*, 2010 ▸; Ding *et al.*, 2009 ▸) and the results of recent compact FEL studies (Bonifacio *et al.*, 1984 ▸; Saleh & Al-Aish, 2023 ▸; Galletti *et al.*, 2024 ▸).

### 4.4. Bit rate estimation from pulse duration

**Figure 5:**
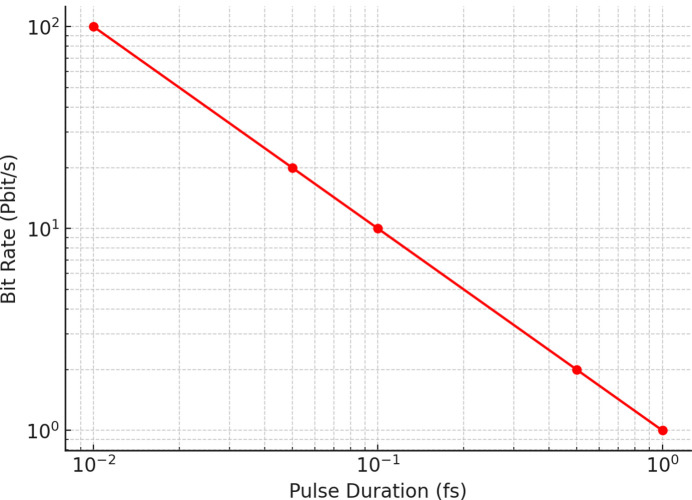
The relation between pulse duration versus bit rate.

The relationship between achievable bit rate and FEL pulse duration is illustrated in Fig. 5 ▸. Femtosecond pulses allow data rates of the order of 10^15^ bits s^−1^, while attosecond-scale pulses extend this capacity to nearly 10^16^ bits s^−1^. These findings confirm the potential of FELs to support petabit-per-second transmission rates, consistent with recent attosecond FEL demonstrations (Xiang & Stupakov, 2009 ▸; Ding *et al.*, 2009 ▸; Yan *et al.*, 2024 ▸).

### 4.5. SNR

**Figure 6:**
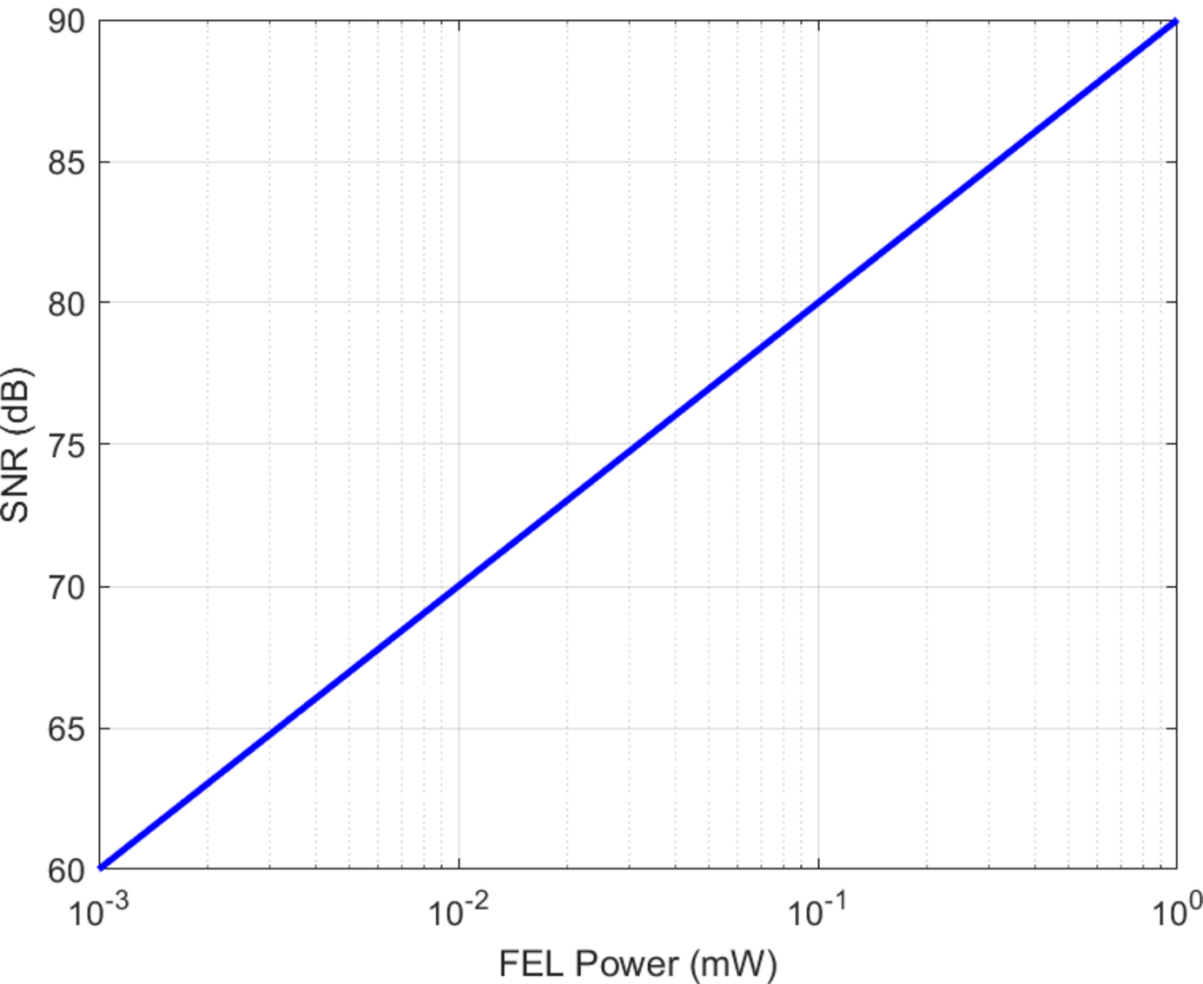
SNR as a function of FEL output power in a free-space optical communication channel.

Fig. 6 ▸ presents the SNR dependence on FEL output power in a free-space optical channel. Even at low power levels (microwatts to milliwatts) the SNR remains above 80 dB. Such performance is critical for both classical high-speed links and QKD, where secure information transfer requires suppression of noise (Vahlbruch *et al.*, 2008 ▸; Saleh & Al-Aish, 2023 ▸; Jiang *et al.*, 2024 ▸).

### 4.6. Overall discussion

The results displayed in Figs. 1 ▸–6 demonstrate that compact FELs achieve two essential requirements needed for beyond-6G and quantum-secure communication systems through their ability to produce broad spectral tuning capabilities and their capacity to increase gain performance in accordance with current requirements and their production of ultrashort pulses and their generation of high SNR output. The results confirm the theoretical framework while demonstrating compatibility with the recent development of smaller FEL designs (Pellegrini *et al.*, 2016 ▸; England *et al.*, 2014 ▸; Niedermayer *et al.*, 2021 ▸; Morel *et al.*, 2025 ▸).

The numerical results which this work presents should be understood as conservative estimates because they rely on small-signal gain formulas. The real-world compact FEL systems will achieve their dominant operational mode through performance of SASE and externally seeded FEL systems in their exponential gain operating range. The power growth in this regime accelerates at a faster rate while reaching higher saturation points and delivering better temporal coherence. The predicted communication performance metrics for this study which include bit-rate SNR and bandwidth should be viewed as minimum thresholds because actual systems will perform at levels which surpass these thresholds.

## 5. Challenges and future perspectives

The study achieved promising theoretical and numerical results but researchers encountered multiple obstacles that must be solved before compact FELs can be used in beyond-6G and quantum communication systems. The challenges that need to be addressed stem from three main areas which include scalability problems, efficiency issues and difficulties with system integration.

### 5.1. Key challenges

*Large-scale infrastructure requirements.* Traditional FEL facilities need multiple advanced accelerator systems, cryogenic components and high-vacuum technologies to cover their operational range which exceeds 100 m (Pellegrini *et al.*, 2016 ▸). The communication networks need compact scalable architectures which cannot function with the current infrastructure system for the existing network.

*Beam quality and stability.* The operational limits of FEL systems depend entirely on the quality of the electron beam which serves as their most critical element. The field of compact accelerator technology faces challenges when trying to produce accelerators which achieve high brightness, low emittance and stable energy spread according to LPAs (Esarey *et al.*, 2009 ▸; Saleh & Al-Aish, 2023 ▸). The fundamental properties which are required for operation to achieve full FEL output coherence and stability need to be present.

*Reliability and repetition rate.* FEL systems require reliable operation because they need strong beam quality for their effective operational performance and system efficiency. The majority of current RF–linac-based FEL systems do not function continuously throughout the day because they need regular maintenance and equipment alignment and system stability testing. The modern telecommunication systems demand reliable operations which the existing operational limits cannot provide. The LPAs offer compact designs but their current operational capacity runs at low repetition rates which the driving laser systems restrict. The average photon flux and data throughput fall under this restriction which represents the main obstacle for executing effective communication systems. The electron beam quality of LPAs matches conventional RF–linac accelerators because it achieves similar performance in energy spread and emittance and long-term stability. The combination of reliability and repetition rate restrictions functions as essential technological obstacles which must be overcome along with the capacity to expand and utilize energy before operational compact FELs can be used in beyond-6G and quantum communication networks.

*Power efficiency.* The compact FEL prototypes generate coherent radiation; however, their energy efficiency issues create operational boundaries which stop their application in continuous and large-scale communication systems (Galletti *et al.*, 2024 ▸; Morel *et al.*, 2025 ▸). The improved efficiency standards enable FEL systems to operate as reliable photonic sources which scientists can use in their actual work environments.

*System integration.* The establishment of compact FELs for terrestrial and satellite communication systems requires solutions that address three specific requirements for packaging needs, thermal management and compatibility with existing photonic devices. The current status of hybrid integration with semiconductor or dielectric photonics technology exists at its initial development stage according to research studies (England *et al.*, 2014 ▸; Jiang *et al.*, 2024 ▸; Niedermayer *et al.*, 2021 ▸).

### 5.2. Miniaturization strategies

The existing obstacles are being addressed through multiple active methods:

*LPAs.* LPAs can produce relativistic electron beams which travel through distances of 1 cm, which allows for a substantial reduction in FEL size requirements according to reference sources (Bonifacio *et al.*, 1984 ▸; Esarey *et al.*, 2009 ▸; Saleh & Al-Aish, 2023 ▸; Morel *et al.*, 2025 ▸).

*Thermionic gun based THz FEL concept.* Scientists can use thermionic electron guns which produce high current electron beams for FEL operation to develop compact THz FELs. Recent research examined electron sources which use thermionic cathodes to produce multi-megavolt output, delivering high peak and average beam power for THz FELs while the study provided performance predictions for small FEL systems (True *et al.*, 2024 ▸). The development of these thermionic gun technologies enables operators to generate high average power output, which provides superior performance compared with typical photocathode or laser-driven systems that use traditional methods.

*Dielectric undulators.* Compact FELs can produce shorter undulator periods through the use of dielectric microstructures instead of magnetic undulators because this method requires less energy to operate than current magnetic undulator systems according to research findings (England *et al.*, 2014 ▸; Jiang *et al.*, 2024 ▸).

*Permanent-magnet technologies.* The development of advanced permanent magnets which operate at high magnetic fields enables scientists to build smaller and lighter undulator systems, which drives progress in FEL miniaturization (Pellegrini *et al.*, 2016 ▸; Niedermayer *et al.*, 2021 ▸).

*Hybrid photonic–electronic systems.* The combination of FEL modules and on-chip photonic circuits will enhance system packaging and stability, while the system will remain compatible with existing communication networks (Jiang *et al.*, 2024 ▸; Niedermayer *et al.*, 2021 ▸).

### 5.3. Future perspectives

Research must address the main obstacles which obstruct the development of compact FELs as essential technologies for future communication systems:

(i) Beyond-6G communication links. FELs enable data transmission at petabit-per-second speeds because they support wide-ranging spectral usage (Akyildiz *et al.*, 2020 ▸; Zhang *et al.*, 2019 ▸; Yan *et al.*, 2024 ▸).

(ii) Quantum-secure communication. The combination of high coherence and ultrashort pulse generation in FELs serves as ideal sources for QKD and entanglement-based protocols (Shapiro, 2009 ▸; Vahlbruch *et al.*, 2008 ▸).

(iii) Satellite systems use compact FEL modules to establish ultra-long-distance communication links which maintain high-fidelity performance throughout their range of operation (Morel *et al.*, 2025 ▸; Al-Aish & Saleh, 2023 ▸).

(iv) Multi-band optical networks. FELs enable adaptive network systems to establish dynamic multi-band allocation through their capability to cover THz optical and X-ray spectral ranges (Feng & Deng, 2020 ▸; England *et al.*, 2014 ▸).

## 6. Conclusion

The authors evaluated whether compact FELs could function as disruptive technologies which would enable new communication methods for beyond-6G and quantum systems. The authors created a theoretical framework that allowed them to examine resonance conditions, gain dynamics, coherence properties, pulse-duration-limited bit rates, and SNR characteristics of the system. MATLAB simulations were used to confirm the accuracy of the models while the FEL performance was tested through various compact design parameters. The results demonstrate that compact FELs offer:

(i) Broad spectral tunability from THz to X-ray regimes through adjustment of electron energy and undulator period.

(ii) High small-signal gain with a clear dependence on electron beam current enabling strong amplification for communication channels.

(iii) Petabit-per-second bit rates which can be achieved through attosecond-scale pulses exceed the maximum output of current optical sources.

(iv) Exceptional SNR performance delivery which maintains over 80 dB at minimal output levels to provide both dependable operation and quantum encryption capabilities.

The research demonstrates that compact FELs can produce ultrashort pulse generation and maintain high coherence while operating across various bands to become essential technologies for future ultra-fast quantum-secure networks. The current beam technology environment encounters three primary obstacles which require researchers to develop better beam quality, greater efficiency, and complete system integration abilities. The creation of LPAs and dielectric undulators and permanent-magnet designs and hybrid photonic–electronic integration systems enables the development of compact devices which operate effectively in actual work environments. The existing research base of compact FELs will continue to develop because scientists will use these systems as research tools to create essential elements for cutting-edge 6G systems and quantum communication networks. The theoretical base of the research establishes will support upcoming experimental work to create scalable high-performance FEL systems for global communication network applications.
